# Comparison of in-hospital mortality risk prediction models from COVID-19

**DOI:** 10.1371/journal.pone.0244629

**Published:** 2020-12-28

**Authors:** Ali A. El-Solh, Yolanda Lawson, Michael Carter, Daniel A. El-Solh, Kari A. Mergenhagen

**Affiliations:** 1 VA Western New York Healthcare System, Buffalo, New York, United States of America; 2 Department of Medicine, Division of Pulmonary, Critical Care, and Sleep Medicine, Jacobs School of Medicine, University at Buffalo, Buffalo, New York, United States of America; 3 Department of Epidemiology and Environmental Health, School of Public Health, University at Buffalo, Buffalo, New York, United States of America; Azienda Ospedaliero Universitaria Careggi, ITALY

## Abstract

**Objective:**

Our objective is to compare the predictive accuracy of four recently established outcome models of patients hospitalized with coronavirus disease 2019 (COVID-19) published between January 1^st^ and May 1^st^ 2020.

**Methods:**

We used data obtained from the Veterans Affairs Corporate Data Warehouse (CDW) between January 1^st^, 2020, and May 1^st^ 2020 as an external validation cohort. The outcome measure was hospital mortality. Areas under the ROC (AUC) curves were used to evaluate discrimination of the four predictive models. The Hosmer–Lemeshow (HL) goodness-of-fit test and calibration curves assessed applicability of the models to individual cases.

**Results:**

During the study period, 1634 unique patients were identified. The mean age of the study cohort was 68.8±13.4 years. Hypertension, hyperlipidemia, and heart disease were the most common comorbidities. The crude hospital mortality was 29% (95% confidence interval [CI] 0.27–0.31). Evaluation of the predictive models showed an AUC range from 0.63 (95% CI 0.60–0.66) to 0.72 (95% CI 0.69–0.74) indicating fair to poor discrimination across all models. There were no significant differences among the AUC values of the four prognostic systems. All models calibrated poorly by either overestimated or underestimated hospital mortality.

**Conclusions:**

All the four prognostic models examined in this study portend high-risk bias. The performance of these scores needs to be interpreted with caution in hospitalized patients with COVID-19.

## Introduction

Since the first reported case of COVID-19 in Wuhan, China, at the end of 2019, COVID-19 has rapidly spread throughout the globe shattering world economy and traditional way of life [1]. As of August 1, 2020, more than 17 million laboratory-confirmed cases had been reported worldwide. The number of infected individuals has surpassed that of SARS and MERS combined. Despite the valiant public health responses aimed at flattening the curve to slow the spread of the virus, more than 675000 people have died from the disease [2].

Numerous prognostic models ranging from rule based scoring systems to advanced machine learning models have been developed to provide prognostic information on patients with COVID-19 [3]. Such information is valuable both to clinicians and patients. It allows healthcare providers to stratify treatment strategy and plan for appropriate resource allocation. As for patients, it offers valuable guidance when advance directives are to be implemented. However, initial description of these prognostic models has been based on patients from a localized geography and time frame. These evaluations may thus be limited in scope of their predictability as concerns have been raised about the applicability of such models when patient demographics change with geography, clinical practice evolves with time, and when disease prevalence varies with both [4, 5]. In response to the call for sharing relevant COVID-19 research findings, many of these models have been published in open access forums before undergoing a peer review. The quality of these models are further compromised by the relatively small sample size both in derivation and validation [6]. Recently, Wynants and colleagues [7] conducted a systematic review of COVID-19 models developed for predicting diagnosis, progression, and mortality from the infection. All models reviewed were at high risk of bias because of improper selection of control patients, data overfitting, and exclusion of patients who had not experienced the event of interest by the end of the study. Besides, external validation of these models was rarely performed. In the present study, we sought to examine the external validity of four scoring models that have shown excellent precision for predicting hospitalization outcome from COVID-19 [8–10].

## Methods

### Patients

We used data from the Veterans Affairs Corporate Data Warehouse (CDW) of all patients tested positive on the reverse transcriptase polymerase chain reaction assay for severe acute respiratory syndrome coronavirus 2 (SARS-CoV-2) between January 1^st^, 2020 and May 1^st^ 2020. Data were extracted from CDW using structure query language (SQL) with pgAdmin4 PostgreSQL 9.6 on July 16, 2020. The de-identified database contained data of demographic information, laboratory values, treatment processes, and survival data. We excluded patients who had a length of stay <24 hours, who lacked vital signs or laboratory data, and who were transferred to or from another acute care facility (because we could not accurately determine the onset or subsequent course of their illness). The median time between the date the case index tested positive for COVID-19 and the date of discharge (whether alive or dead) was referred to as the median follow-up. All data analysis was done on the VA Informatics and Computing Infrastructure workspace (VINCI). Access of the CDW for research was approved by the Institutional Review Board of the VA Western New York Healthcare System. Because the study was deemed exempt, informed consent was not required.

### Missing data

Demographic and comorbidity data contained almost no missing data. However, many baseline laboratory values had up to 20% missing data. When data are missing at random, statistical methods such as multiple imputation give less biased and realistic results compared with complete case analysis [11]. However, the ordering of a laboratory test is likely driven by factors that make assumptions underlying multiple imputation inaccurate. In the absence of a standardized method to address missing data under these conditions, we have adopted the following approach: Missing data were imputed with the centered mean. A dummy variable (called also an indicator variable) is added to the statistical model in order to indicate whether the value for that variable is available or not [12]. When using the indicator method to handle missing covariate data, the value for the missing variable is set to 1, otherwise the value is set to 0. Then, both the primary variable and the indicator variable are entered into the regression model to predict the intended outcome. Then, both the primary variable and missingness indicator were evaluated in a mixed-effects logistic regression model and the primary mean imputed variable was considered.

### External validation of risk models

We conducted initially a search strategy using PubMed and Medline databases between January 1st, 2020 and May 1^st^, 2020. The literature was done using the following keywords in combination: 1) (COVID-19 OR SARS-CoV-2 OR 2019-nCoV) AND 2) (Mortality OR Death) AND 3) (Predictive model OR Scoring system) (“S1 Table” in S1 File). Inclusion criteria were: 1) English-written peer reviewed studies; 2) hospitalized patients with COVID-19; 3) prognostic models for predicting in-hospital mortality; and 4) sample size of no less than 100. Exclusion criteria included duplicate studies and lack of access to full documents. Studies identified by the search strategy were reviewed by title and abstract. Screening was conducted by two independent investigators (YL and DES). Any disagreements were resolved by consensus. Fifteen studies were identified. Two were concise reviews leaving 13 studies for further evaluation. Four prognostic models were selected based on availability of the predictive parameters in the CDW [8–10, 13]. For each predictive model, we replicated the methods used by the original authors to calculate the predicted hospital mortality from COVID-19. The main outcome of interest was in-hospital mortality.

We incorporated the Transparent Reporting of a multivariable prediction model for Individual Prognosis or Diagnosis (TRIPOD) principles for validating each of the selected predictive models [14]. The risk of bias for each predictive model was evaluated by the Prediction model Risk Of Bias Assessment Tool (PROBAST) described by Moons and colleagues [15].

### Statistical analysis

The normality of continuous variables was assessed using the Kolmogorov–Smirnov test. Continuous variables with and without normal distribution were reported as mean (standard deviation (SD)) and median (interquartile range (IQR)), respectively. Categorical variables were presented as number (percentage). Continuous variables with or without normal distribution between survivors and non-survivors were compared using t-test and Mann–Whitney U test, respectively. Comparisons of categorical variables were performed using Chi squares tests.

Receiver operating characteristic (ROC) curves were drawn for each model by plotting sensitivity versus one minus specificity. The area under the receiver operating characteristic curve (AUC) was used to evaluate the discriminatory capacity of the selected models [16]. An ideal discrimination produces an AUC of 1.0, whereas discrimination that is no better than chance produces an AUC of 0.5. Based on a rough classifying system, AUC can be interpreted as follows: 90–100 = excellent; 80–90 = good; 70–80 = fair; 60–70 = poor; 50–60 = fail [17]. Pair-wise comparison of the area under the ROC curve for each model was performed according to the method described by Hanley and McNeil [16]. If *P* is less than the conventional 5% (*P* < .05), the compared areas are considered statistically different. Calibration was assessed with the Hosmer–Lemeshow goodness-of-fit χ^2^ estimates by grouping cases into deciles of risk [18]. The method involves sorting the predictive probabilities of death in ascending order and dividing the total number of cases into 10 equally distributed subgroups or deciles. Calibration plots were provided to show the relationship between model-based predictions of mortality and observed proportions of mortality using the loess algorithm [19]. The non-parametric bootstrapping method was used to calculate the 95% confidence intervals (CIs) of both discrimination and calibration estimates [20]. These CIs were reported using the percentile method, or bias corrected method if the estimation bias was greater than 25% of the standard error [21]. All analyses were performed using STATA 15.0 (STATA Corp). A *P*-value less than .05 was considered statistically significant.

## Results

A total of 1634 patients were hospitalized for COVID-19 between January 1, 2020 and May 1, 2020. The majority of patients were male (95%) with 47% identified as Caucasian, 43% as African American, and 10% as Latino. Fever (65%), dyspnea (41%), and cough (32%) were the three most common manifestations at hospital admission. The mean age of the cohort was 68.8±13.4 years. Fifty percent of the group had three or more comorbidities. Hypertension was the most common comorbidity, followed by hyperlipidemia, and heart disease. The median time from illness onset to admission was 7.8 days (interquartile range 1.0–14.2). Of the 817 patients treated in the intensive care units, 478 (59%) required invasive mechanical ventilation. Overall, 73.8% received at least one antibiotic treatment during their hospital stay. Almost half of the patients had received azithromycin and/or hydroxychloroquine. After a median follow-up of 58 (IQR, 50–68) days, there were 475 deaths (overall mortality, 29%) for a mortality rate of 12 (95%CI, 11–12) per 1000 patient-days.

The clinical characteristics of survivors and non-survivors of the CDW cohort are depicted in “Table 1”. In univariate analysis, age, current tobacco smoker, high burden of comorbidities, lymphopenia, thrombocytopenia, liver function abnormalities, and elevated procalcitonin and D-dimer levels were associated with mortality. Compared with survivors, non-survivors were more likely to receive vasopressors, to require mechanical ventilation, and to develop complications including acute respiratory distress syndrome, acute renal failure, and septic shock.

**Table 1:** Comparison of baseline characteristics and treatment between survivors and non-survivors of the external validation group.

	Study population N = 1,634	Missing observation (%)	Survivors N = 1,159	Non-survivors N = 475	P value
Age, years	68.8±13.4	0	66.1±13.5	75.6±10.6	<0.001
Sex, n (%)		0			0.004
Male	1553 (95)		1,090 (94)	463 (97)	
Female	81 (5)		69 (6)	12 (3)	
Race, n (%)		0			0.24
Caucasians	772 (47)		562 (48)	210 (44)	
Black	699 (43)		481 (42)	218 (46)	
Latinos	163 (10)		116 (10)	47 (10)	
BMI, kg/m^2^	28 (24–33)	1.0	27 (23–32)	29 (25–33)	<0.001
Current smoker, n(%)	171 (10)	0	104 (9)	67 (14)	0.002
Comorbidities, n (%)					
COPD	404 (25)	0	261 (23)	143 (30)	0.001
Diabetes mellitus	801 (49)	0	544 (47)	257 (54)	0.008
Hypertension	1208 (74)	0	839 (72)	369 (78)	0.03
CAD	461 (28)	0	290 (25)	171 (36)	<0.001
Heart failure	299 (18)	0	177 (15)	122 (26)	<0.001
Chronic renal failure	111 (7)	0	74 (6)	37 (8)	0.31
CVD	85 (5)	0	49 (4)	36 (41)	0.006
Liver cirrhosis	65 (4)	0	47 (4)	18 (4)	0.8
HIV infection	32 (2)	0	22 (2)	10 (2)	0.784
Charlson Comorbidity Index	3 (1–6)	0	2 (1–5)	4(2–7)	<0.001
ICU admission	817 (50)	0	462 (39)	355 (74)	<0.001
Signs and Symptoms, n(%)					
Fever	1064 (65)	0	764 (66)	300 (63)	0.29
Cough	530 (32)	0	414	116 (24)	<0.001
Dyspnea	666 (41)	0	463	203 (43)	0.29
Fatigue	251 (15)	0	177	74 (16)	0.87
Diarrhea	165 (10)	0	132	33 (7)	0.007
Laboratory results, n(%)					
WBC, x10^9^/L	6.2 (4.8–8.4)	1.3	6 (4.7–8)	6.7 (5.1–9.7)	<0.001
Lymphocytes, x10^9^/L	0.88 (0.58–1.25)	1.9	0.92 (0.62–1.3)	0.77 (0.49–1.07)	<0.001
Hemoglobin, g/L	13.25 (11.7–14.6)	9.7	13.6 (11.9–14.7)	12.9 (11.4–14.5)	0.2
Platelets, x10^9^/L	179 (128–230)	5.8	193 (150–246)	161 (102–221)	<0.001
Creatinine	1.3 (1.0–1.9)	0.1	1.21 (0.95–1.66)	1.52 (1.1–2.5)	<0.001
AST, U/L	39 (26–58)	6.7	37 (25–55)	45 (29–71)	<0.001
ALT, U/L	29 (19–44)	6.7	29 (1–44)	29 (19–46)	0.87
Procalcitonin, ng/mL	0.17 (0.08–0.46)	9.1	0.13 (0.07–0.3)	0.32 (0.13–1.21)	<0.001
D-Dimer, ug/mL	215 (1.57–617)	18.7	183.5 (1.2–524.0)	297.5 (2.7–868.5)	<0.001
Treatment, n(%)					
Mechanical ventilation	478 (29)	0	190 (16)	288 (61)	<0.001
Remdesivir	86 (5)	0	64 (6)	22 (5)	0.46
Hydroxychloroquine	747 (46)	0	493 (43)	254 (53)	<0.001
Interleukin6-inhibitor	261 (16)	0	182 (16)	79 (17)	0.64
Vasopressors	374 (23)	0	144 (12)	230 (48)	<0.001
Complications					
ARDS	263 (16)	0	115 (9.9)	148 (31)	<0.001
Acute renal failure	813 (49)	0	472 (41)	341 (72)	<0.001
Septic shock	530 (32)	0	273 (24)	257 (54)	<0.001

ARDS = Acute Respiratory Distress Syndrome

AST = Aspartate transaminase

ALT = Alanine transaminase

BMI = Body Mass Index

CAD = Coronary Artery Disease

CVD = Cerebrovascular Disease

COPD = Chronic Obstructive Lung Disease

**Fig 1:**
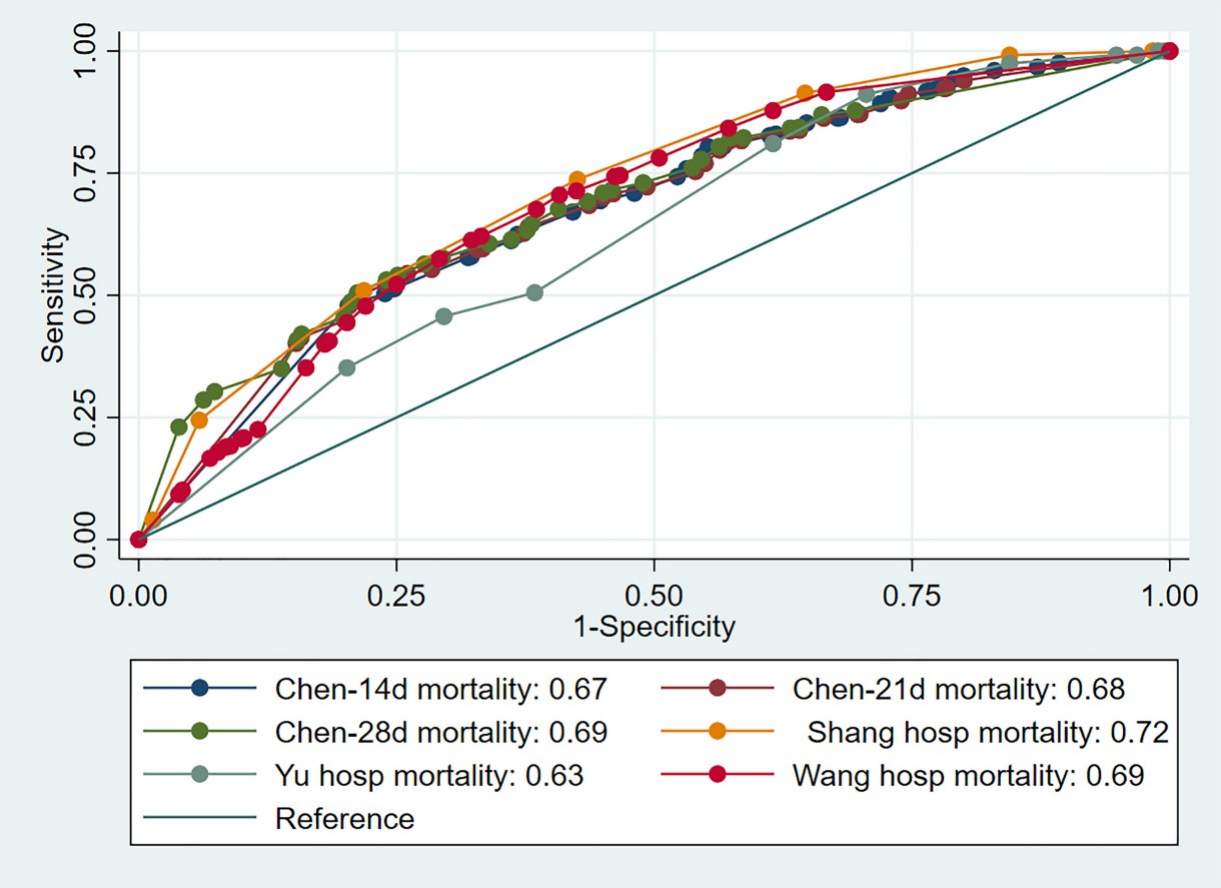
Receiver-operating characteristics curves of the prognostic models.

A summary of models methodology is depicted in “S2 Table” in S1 File. “Table 2” shows the independent risk variables and corresponding odds ratios of the four prognostic models. All four models were classified as overall high ROB either because of flawed methods of data analysis pertaining to handling of missing data or lack of validation cohort “S3 Table” in S1 File. The predictive performances of the four models on the CDW cohort are presented in “Table 3”. The AUCs indicate inferior discriminative power across all models compared to the AUCs obtained by the derivation cohorts. Pair-wise comparisons of the AUCs were performed by using the method described by Hanley and McNeil [22] “Table 4”. Overall the best discrimination was obtained by the scoring model proposed by Shang et al. [9] which attained significance with respect to Chen et al. [8] and Yu et al. [10] models (AUCs 0.72 (95% CI 0.69–0.74) versus 0.68 (0.66–0.70) and 0.63 (95% CI 0.60–0.66); respectively) “Fig 1”. The least discriminatory model was the model described by Yu et al. [10] with an AUC of 0.63 (95% CI 0.60–0.66).

**Table 2:** Odds ratios and 95% confidence intervals of the components of the three predictive models.

Parameters	Chen et al. [8]	Shang et al. [9]	Yu et al. [10]	Wang et al. [13]
Sample Size	1590	452	1464	296
Age, years				1.11 (1.05–1.17)
<65	1.0			
65–74	3.43 (1.24–9.5)			
≥75	7.86 (2.44–25.35)			
Age, years				
<60		1.0		
60–75		1.82 (0.41–8.17)		
>75		15.07 (2.27–99.78)		
Age, years				
<65			1.0	
≥65			2.11 (1.39–3.21)	
Sex				
Female			1.0	
Male			2.02 (1.37–2.99)	
Hypertension				1.82 (0.5–6.63)
Diabetes mellitus			2.52 (1.62–3.94)	
CAD	4.28 (1.14–16.13)	5.61 (1.39–22.62)		3.04 (0.45–20.74
CVA	3.1 (1.07–8.94)			
Dyspnea	3.96 (1.42–11.0)			
AST, U/L				
>40	2.2 (1.1–6.73)			
PCT, ng/ml				
>0.5	8.72 (3.42–22.28)			
>0.15		20.74 (5.14–83.75)		
≥0.05			3.13 (2.02–4.84)	
Lymphocytes, %				
<8%		3.66 (1.01–13.38)		
Lymphocytes, x10^9^/L				
<1.1			1.45 (0.98–2.15)	
D-dimer, ug/ml				
>0.5		4.45 (1.37–14.51)		

**Table 3:** Summary of the discrimination and calibration performance for each model.

	AUC_d_	AUC_v_	HLχ^2^	HL_(p)_
Chen et al. [8]	0.91 (0.85–0.97)	0.68 (0.66–0.70)		
14d mortality		0.67 (0.64–0.70)	377.3	<0.001
21d mortality		0.68 (0.65–0.71)	1015.8	<0.001
28d mortality		0.69 (0.66–0.72)	1805.3	<0.001
Shang et al. [9]	0.92 (0.87–0.97)	0.72 (0.69–0.74)	44.3	<0.001
Yu et al. [10]	0.77 (0.73–0.81)	0.63 (0.60–0.66)	124.4	<0.001
Wang et al. [13]	0.88 (0.79–0.94)	0.69 (0.66–0.72)	62.8	<0.001

AUC_d_ = Area under the curve of the derivation model; AUC_v_ = Area under the curve of the validation model; HL = Hosmer Lemeshow

**Table 4:** Pair-wise discrimination model comparison.

	Chen (14d)		Chen (21d)		Chen (28d)		Shang		Yu	
	Diff	*p*	Diff	*p*	Diff	*p*	Diff	*p*	Diff	*p*
Chen (14d)										
Chen (21d)	0.003	0.87								
Chen (28d)	0.015	0.48	0.011	0.56						
Shang	0.044	0.03	0.041	0.04	0.029	0.12				
Yu	0.047	0.03	0.051	0.01	0.063	0.002	0.09	<0.001		
Wang	0.016	0.46	0.012	0.54	0.0006	0.97	0.028	0.13	0.063	0.001

**Fig 2:**
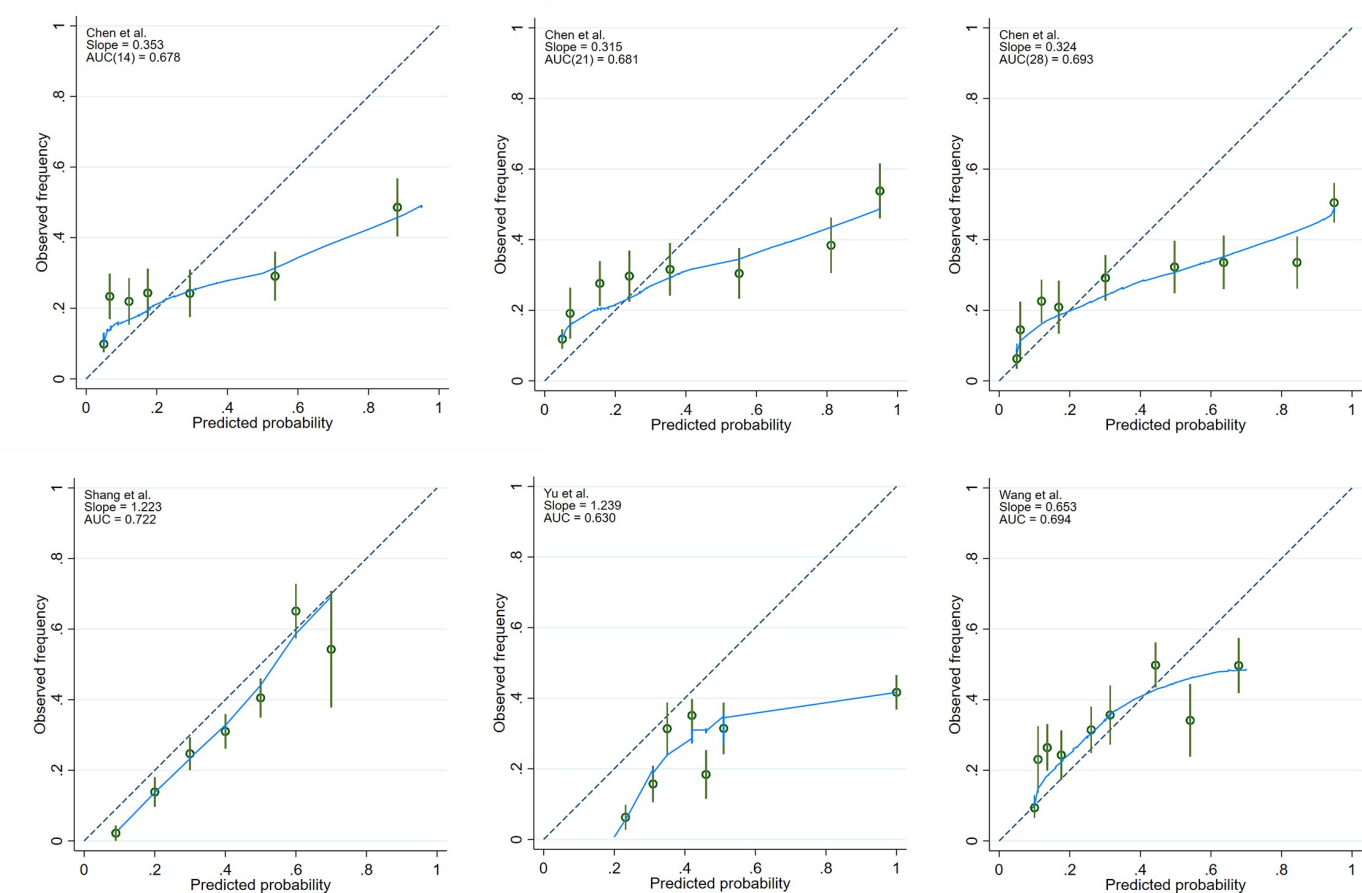
Calibration plots. The green circles denote point estimates and the green vertical lines 95% confidence intervals for risk groups. Fewer than 10 groups in a plot indicate absence of cases in decile risk groups. The dashed line represents a perfect agreement between observed and expected mortality estimates. The blue line indicates the fitted loess curve. (A-C) represent the calibration plots generated from Chen et al. (D) represents the calibration plot generated from Shang et al. (E) denotes the calibration plot generated from Yu et al. (F) illustrates the calibration plot generated from Wang et al.

The Hosmer–Lemeshow goodness-of-fit test reveals poor calibration (p < 0.05) for all the models “Table 3”. Calibration was further explored by plotting the observed to expected frequency of death for each quintile of every model “Fig 2”. The Chen et al. model [8] showed a departure from expected risks at the tail of risk distribution for each of the three endpoints selected (14, 21, and 28 days predicted mortality) “Fig 2A–2C”. The predictions overestimated the probability of death for high risk patients. This was also the case for the model by Yu et al. [10] “Fig 2E”. In contrast, the model by Wang et al. [13] underestimated the probability of death for low risk patients and overestimated it for high risk patients “Fig 2F” while Shang et al. model [9] consistently overestimated mortality risk across the range of total scores “Fig 2D”.

## Discussion

This is, to our knowledge, the first study to evaluate and externally validate risk prediction models of in-hospital mortality from COVID-19 in a large cohort. Our results showed that external validation of all four selected scores was not commensurate with the performance observed in the primary derivation cohorts underscoring that model evaluation can generally be generalizable only when the model has been tested in a separate cohort exposed to similar risk pressure.

With the rapid spread of COVID-19, healthcare providers struggle to institute clinical strategies aiming at optimizing outcomes and reducing resource consumption. In response, more than two dozen prediction models have been destined for publications in just over 12 weeks period since COVID-19 was declared a pandemic by the WHO [3]. Many of the prediction models were developed as simplified scoring system or nomograms. Despite the excellent predictive accuracy shown in the initial derivation, the validity of these models has not been confirmed independently. Based on our observations, the performance of these prognostic systems varied in their ability to discriminate between survivors and non-survivors and were labeled overall either fair or poor in contrast to their original designation as excellent or good. We should point out that the four models originated from mainland China that was initially hard hit by the pandemic. With the large disparity in medical resources among the Chinese provinces [23], the expected models can only be accurate under the same clinical setting the model was derived under. As such, the risk prediction models developed in a different geographic setting can be less accurate in providing risk-adjusted outcomes when applied externally [24].

Various statistical and clinical factors may lead to a prognostic model to perform poorly when applied to other cohorts [25]. First, the models presented in this study are parsimonious, making a variety of assumptions in order to simplify applicability and avoid overfitting the limited and often incomplete data available. Even when these predictive models being constructed with similar variables such as age, presence of comorbidities, and laboratory values (procalcitonin, C-reactive protein, or D-dimer), the thresholds selected for each of these variables vary significantly for a given geographic locality [26]. Second, these models have several sources of uncertainty, including the definition of parameters entered into the final model, differences in handling missing data, and most importantly, non-comparable traits (genetic diversity), which can weaken model prognostication and lessen its discrimination accuracy [27, 28].

Even when discrimination can be useful for generic risk stratification, the observed poor calibration underlines the fact that the applicability of these prognostic scoring models to heterogeneous systems of health care delivery dissimilar to the derivation cohorts may not be feasible. The four prognostic models showed shortcomings with regard to calibration, tending to over-predict or under-predict hospital mortality. This may partly reflect the inclusion criteria of the sample–in which, for example, do not resuscitate patients were not included–and improvements in care (e.g. timing to transfer to ICU or prone position in management of ARDS) since the models were first developed. A relevant factor in explaining the divergence in performance accuracy is that the time from onset of illness to admission was not similar among all cohorts. Wang and colleagues [13] reported the shortest interval of a median of 5.0 days for survivors and 6.8 days for non-survivors while Yu and coworkers [10] reported a median of 10.0 days for both the survivors and non-survivors. Our interval was comparable to the study of Shang and colleagues [9] which may explain the higher performance of that study using the CDW cohort.

It could be argued also that our CDW cohort consisted of predominantly male, Caucasian and African American patients with multiple comorbidities which are different from the patient demographics in the original training dataset and, as such, may impose significant strain on the accuracy of the risk estimates. Age-standardized mortality in men was shown to be almost double compared to that of women across all age groups [29]. Reports have similarly suggested a disproportionate mortality rates among Black and Latino residents compared with their proportion of the US population. Age and population adjusted Black mortality was reported more than twice that for Whites [30, 31]. Accordingly, the predictive models might be expected to give different predictions of mortality risk in our validation cohort. While this may cause prognostic systems to underestimate the mortality rate at the lower end of the calibration curve, only two out of the four tested models exhibited this pattern. Multiple studies have demonstrated a decreasing ratio of observed mortality to expected mortality with time [32, 33]. Changing risk profiles, advance treatment modalities, and changes in the association of risk factors with outcomes can all contribute to poor calibration. Given that the CDW cohort overlaps the time period during which the four models were constructed, we cannot attribute the failure of the Hosmer-Lemeshow tests to this phenomenon [34]. Consideration of other variables, such as severity of comorbid diseases, lifestyle habits (smoking or alcohol intake), and prescribed treatments may improve the predictive accuracy of these models. Alternatively, an ensemble learning model [35] which uses multiple decision-making tools can be implemented to produce a more accurate output [36].

Our study has its own strengths but also several limitations. The systematic nature of the model identification, the large sample size in which the models were validated and the opportunity to compare the performance among the predictive models are all substantial strengths. Conversely, at the time our analysis was conducted, the number of COVID-19 cases was relatively small compared to the most recent statistics of veterans infected with COVID-19. This limits the precision in re-estimating the baseline prevalence of the disease, which may have hampered the calibration performance of the model. However, CDW is undergoing continuous update and re-conducting this validation in the large expanded cohort may mitigate some of these issues related to selection bias. Such validations in large datasets have been advocated to ensure developed prediction models are fit for use in all intended settings [37]. Finally, while previous studies have shown that physicians usually overestimate patients’ mortality [38], there is limited evidence so far to suggest that prognostic models represent a superior solution when their performance in actual clinical practice is taken into consideration.

## Conclusions

In conclusion, predictions arising from risk models applied to cohorts drawn from a different distribution of patient characteristics should not be adopted without appropriate validation. The variability in predicted outcomes as we have documented in this analysis highlights the challenges of forecasting the course of a pandemic during its early stages [7]. To achieve a more robust prediction model, the focus should be placed on developing platforms that enable deployment of well-validated predictive models and prospective evaluation of their effectiveness. We are actively engaged in pursuing these objectives at the Veterans Affairs.

## Supporting information

S1 File (DOCX)
