# Supplementary material for: Comparison of in-hospital mortality risk prediction models from COVID-19
Source: PLoS One. 2020 Dec 28;15(12):e0244629. doi: 10.1371/journal.pone.0244629 (PMC7769558; doi:10.1371/journal.pone.0244629)
Supplement: S1 File — (DOCX) [file pone.0244629.s001.docx]

**COMPARISON OF IN-HOSPITAL MORTALITY RISK PREDICTION MODELS FROM COVID-19**

S1 File

**S1 Table. Search Strategy**

| #1 | COVID-19 |
| --- | --- |
|  | "severe acute respiratory syndrome coronavirus 2"[Supplementary Concept] OR "severe acute respiratory syndrome coronavirus 2"[All Fields] OR "ncov"[All Fields] OR "2019-nCoV"[All Fields] OR "COVID-19"[All Fields] OR "SARS-CoV-2"[All Fields] |
| #2 | **Mortality OR Death** |
|  | "mortality"[MeSH Terms] OR "mortality"[All Fields] OR "mortalities"[All Fields] OR "mortality"[Subheading] OR "death"[MeSH Terms] OR "death"[All Fields] OR "deaths"[All Fields] |
| #3 | **Predictive Model OR Scoring System** |
|  | "predict"[All Fields] OR "predictabilities"[All Fields] OR "predictability"[All Fields] OR "predictable"[All Fields] OR "predictably"[All Fields] OR "predicted"[All Fields] OR "predicting"[All Fields] OR "prediction"[All Fields] OR "predictions"[All Fields] OR "predictive"[All Fields] OR "predictively"[All Fields] OR "predictiveness"[All Fields] OR "predictives"[All Fields] OR "predictivities"[All Fields] OR "predictivity"[All Fields] OR "predicts"[All Fields] OR "model"[All Fields] OR "model's"[All Fields] OR "modeled"[All Fields] OR "modeler"[All Fields] OR "modeler's"[All Fields] OR "modelers"[All Fields] OR "modeling"[All Fields] OR "modelings"[All Fields] OR "modelization"[All Fields] OR "modelizations"[All Fields] OR "modelize"[All Fields] OR "modelized"[All Fields] OR "modelled"[All Fields] OR "modeller"[All Fields] OR "modellers"[All Fields] OR "modelling"[All Fields] OR "modellings"[All Fields] OR "models"[All Fields] OR "score"[All Fields] OR "score's"[All Fields] OR "scored"[All Fields] OR "scores"[All Fields] OR "scoring"[All Fields] OR "scorings"[All Fields] OR "drug delivery systems"[MeSH Terms] OR ("drug"[All Fields] AND "delivery"[All Fields] AND "systems"[All Fields]) OR "drug delivery systems"[All Fields] OR "system"[All Fields] OR "system's"[All Fields] OR "systems"[All Fields] |

**S2 Table. Methodology of the COVID-19 predictive models for hospital mortality**

|  | Chen et al.(1) | Shang et al.(2) | Yu et al.(3) | Wang et al.(4) |
| --- | --- | --- | --- | --- |
| Design | Retrospective | Retrospective | Retrospective | Observational |
| Site(s) | 575 hospitals throughout China | Zhongnan Hospital, Wuhan, China  Leishenshan Hospital, Wuhan, China | Tongji Hospital, Wuhan, China | First Peoples’ Hospital, Wuhan, China  Union Hospital, Wuhan, China |
| Recruitment period | -1/31/2020 | 1/1/2020-3/27/2020 | 1/14/2020-2/28/2020 | 1/7/2020-2/20/2020 |
| Derivation cohort (N) | 1,590 | 113 | 1,663 | 296 |
| Validation cohort (N) | NA | 339 | NA | 44 |
| Participants | Review of hospital medical records | Review of hospital medical records | Review of hospital medical records | Consecutive hospitalized patients diagnosed with COVID-19 |
| Inclusion criteria | 1) Hospitalized patients with COVID-19 defined by positive PCR; 2) high throughput sequencing of viral genome of specimens obtained from nasal or pharyngeal swab | Consecutive patients with confirmed COVID-19 by PCR | Hospitalized patients with COVID-19 defined by positive PCR | 1) Respiratory tract or blood specimens  positive for COVID-19 by  polymerase chain reaction; 2) virus in respiratory tract  or blood specimens highly homologous with COVID-19 by genetic sequencing; or 3) suspected cases with  imaging features of pneumonia |
| Exclusion criteria | Incomplete medical records | Negative COVID-19 test | Need for continuous hospitalization, transfer to other hospitals | Need for continuous hospitalization, pregnancy, multiorgan dysfunction on admission, missing critical data |
| Model derivation | Backward stepdown regression  (nomogram) | Least absolute shrinkage and selection operator regression (risk score) | Multivariate logistic regression  (risk score) | Multivariate logistic regression  (nomogram) |
| Primary outcome | Mortality | In-hospital mortality | In-hospital mortality | In-hospital mortality |
| Predicted outcome | 14-,21-,28-day survival | Mortality | Mortality | Mortality |
| Handling of missing data | Not included | Multiple imputation | Not included | Not included |
| Model Performance | | | | |
| Discrimination | Concordance index | AUC | AUC | AUC |
| Calibration | Bootstrapping | Hosmer-Lemeshow goodness of fit | Not included | Not included |

*NA= Not available; PCR= polymerase chain reaction; AUC= Area under the curve

**S3 Table. Risk of bias and applicability of COVID-19 outcome prediction models according to PROBAST**

| Study | ROB | | | | Applicability | | | Overall judgment | |
| --- | --- | --- | --- | --- | --- | --- | --- | --- | --- |
|  | Participants | Predictors | Outcome | Analysis | Participants | Predictors | Outcome | ROB | Applicability |
| Chen et al. (1) | **L** | **L** | **L** | **H** | **L** | **L** | **H** | **H** | **H** |
| Shang et al. (2) | **L** | **L** | **L** | **H** | **L** | **L** | **L** | **H** | **L** |
| Yu et al. (3) | **L** | **L** | **L** | **H** | **L** | **L** | **L** | **H** | **L** |
| Wang et al. (4) | **L** | **L** | **L** | **H** | **L** | **L** | **L** | **H** | **L** |

PROBAST=Prediction model Risk of Bias Assessment Tool; ROB= Risk of Bias

L indicates low ROB/low concern regarding applicability

H indicates high ROB/high concern regarding applicability

References

1. Chen R, Liang W, Jiang M, Guan W, Zhan C, Wang T, et al. Risk Factors of Fatal Outcome in Hospitalized Subjects With Coronavirus Disease 2019 From a Nationwide Analysis in China. Chest. 2020;158(1):97-105.

2. Shang Y, Liu T, Wei Y, Li J, Shao L, Liu M, et al. Scoring systems for predicting mortality for severe patients with COVID-19. EClinicalMedicine. 2020;24:100426.

3. Yu C, Lei Q, Li W, Wang X, Liu W, Fan X, et al. Clinical Characteristics, Associated Factors, and Predicting COVID-19 Mortality Risk: A Retrospective Study in Wuhan, China. Am J Prev Med. 2020;59(2):168-75.

4. Wang K, Zuo P, Liu Y, Zhang M, Zhao X, Xie S, et al. Clinical and Laboratory Predictors of In-hospital Mortality in Patients With Coronavirus Disease-2019: A Cohort Study in Wuhan, China. Clin Infect Dis. 2020;71(16):2079-88.
